# Risk stratification of lung adenocarcinoma using a nomogram combined with ferroptosis-related LncRNAs and subgroup analysis with immune and N6-methyladenosine modification

**DOI:** 10.1186/s12920-022-01164-5

**Published:** 2022-01-29

**Authors:** Chen Gao, Ning Kong, Fan Zhang, Tianyu Tang, Jiaying Li, Honglei Ding, Zhichao Sun, Linyu Wu, Maosheng Xu

**Affiliations:** 1 Department of Radiology, The First Affiliated Hospital of Zhejiang Chinese Medical University (Zhejiang Provincial Hospital of Traditional Chinese Medicine), 54 Youdian Road, Hangzhou, China; 2 The First School of Clinical Medicine, Zhejiang Chinese Medical University, Hangzhou, China; 3 Zhejiang Provincial Key Laboratory of Pancreatic Disease, The First Affiliated Hospital, School of Medicine, Zhejiang University, Hangzhou, Zhejiang China; 4 Department of Hepatobiliary and Pancreatic Surgery, The First Affiliated Hospital, School of Medicine, Zhejiang University, Hangzhou, Zhejiang China

**Keywords:** Lung adenocarcinoma, Ferroptosis, N6-methyladenosine, Long noncoding RNA, Prognosis, Biomarker

## Abstract

**Background:**

Determining the prognosis of lung adenocarcinoma (LUAD) is challenging. The present study aimed to identify prognostic ferroptosis-related long noncoding RNAs (FRLs) and construct a prognostic model. Moreover, differential analysis of immune and N6-methyladenosine (m6A)-related genes was systematically conducted.

**Methods:**

A total of 504 patients selected from a dataset from The Cancer Genome Atlas were included. The patients with LUAD were randomly divided into a training group and a test group at a ratio of 1:1. Pearson correlation analysis and univariate Cox regression analysis were used to identify the prognostic FRLs. Then, a prognostic model was constructed from the optimized subset of prognostic FRLs based on the least absolute shrinkage and selection operator (LASSO) algorithm. Subsequently, the receiver operating characteristic (ROC) curve and survival analysis were used to evaluate the performance of the model. The risk score based on the prognostic model was analyzed using Cox regression analysis. Moreover, gene set enrichment analysis and differential analysis of immune- and m6A-related genes were conducted.

**Results:**

After univariate Cox regression analysis and LASSO algorithm analysis, a total of 19 prognostic FRLs were selected to construct the final model to obtain the risk score. The area under the ROC curve of the prognostic model for 1-year, 3-year, and 5-year overall survival (OS) was 0.763, 0.745, and 0.778 in the training set and 0.716, 0.724, and 0.736 in the validation set, respectively. Moreover, the OS of the high-risk group was significantly worse than that of the low-risk group in the training group (*P* < 0.001) and in the test group (*P* < 0.001). After univariate and multivariate Cox regression analysis, the risk score [hazard ratio (HR) = 1.734; *P* < 0.001] and stage (HR = 1.557; *P* < 0.001) were both considered significant prognostic factors for LUAD. A nomogram was constructed based on clinical features and risk score. The expression of 34 checkpoint genes and 13 m6A-related genes varied significantly between the two risk groups.

**Conclusion:**

This study constructed a prognostic model to effectively predict the OS of patients with LUAD, and these OS-related FRLs might serve as potential therapeutic targets of LUAD.

**Supplementary Information:**

The online version contains supplementary material available at 10.1186/s12920-022-01164-5.

## Background

Lung cancer is a malignancy with high mortality and the second highest incidence worldwide in most countries, with approximately 235,760 new lung cancer cases diagnosed and approximately 131,880 related deaths in 2021 [1]. Generally, the five-year survival of patients with lung cancer ranges from 10 to 20% [2]. Lung adenocarcinoma (LUAD) is the subtype of lung cancer with the highest prevalence [3, 4]. Despite advancements in surgery, radiotherapy, chemotherapy, and other advanced therapeutics, the prognosis remains poor due to the high heterogeneity of LUAD [5, 6]. As such, research into the molecular mechanisms and pathogenesis of LUAD requires further research to develop promising therapies.

Ferroptosis is a specific type of cell death that is typically associated with the accumulation of iron and peroxidation and has been identified as a promising intervention in cancer therapeutics to trigger apoptosis of malignancies that are resistant to traditional methods [7–9]. Ferroptosis-related genes (FRGs) is genes that can promote, prevent or indicate the occurrence of ferroptosis, which annotated as drivers, suppressors or markers [10]. Some studies have even noticed the potential role of FRGs in lung cancer development and suppression, but the specific details remain elusive [11–13]. Long noncoding RNAs (lncRNAs) are a type of noncoding RNA sequence measuring approximately 200 nucleotides in length, and these sequences affect several processes, such as cell growth and apoptosis [14]. The regulation of lncRNAs in ferroptosis has been investigated and found to be related to different malignancies, including lung cancer [15, 16]. However, there are only a few studies on the mechanism of how ferroptosis-related lncRNAs (FRLs) act on the occurrence and progression of LUAD. As such, studies investigating the involvement of FRLs in the development of LUAD may be of great value for identifying potential targets for guided treatment. Separately, N6-methyladenosine (m6A) modification is an important method of RNA posttranscriptional modification in most eukaryotic mRNAs and lncRNAs [17, 18]. Many studies have indicated that m6A modification is related to the oncogenesis and progression of malignant tumors, including LUAD, and could regulate ferroptosis [19–22]. Therefore, it is essential to study these m6A modifications to comprehensively understand the involvement of FRLs in the development of LUAD.

With these important markers in mind, we sought to identify the prognostic value of FRLs using bioinformatics and a statistical analysis of data collected from patients with LUAD from The Cancer Genome Atlas (TCGA) dataset. In addition, a prognostic model will be created using an optimistic subset of FRLs to predict the long-term mortality of LUAD patients and used to construct a nomogram for guiding clinical judgment. Subgroup analysis along with immune and m6A modification will be used in conjunction to further determine the performance of the model and the expression of related genes within the study groups.

## Methods

### Datasets and FRGs

**Fig. 1:**
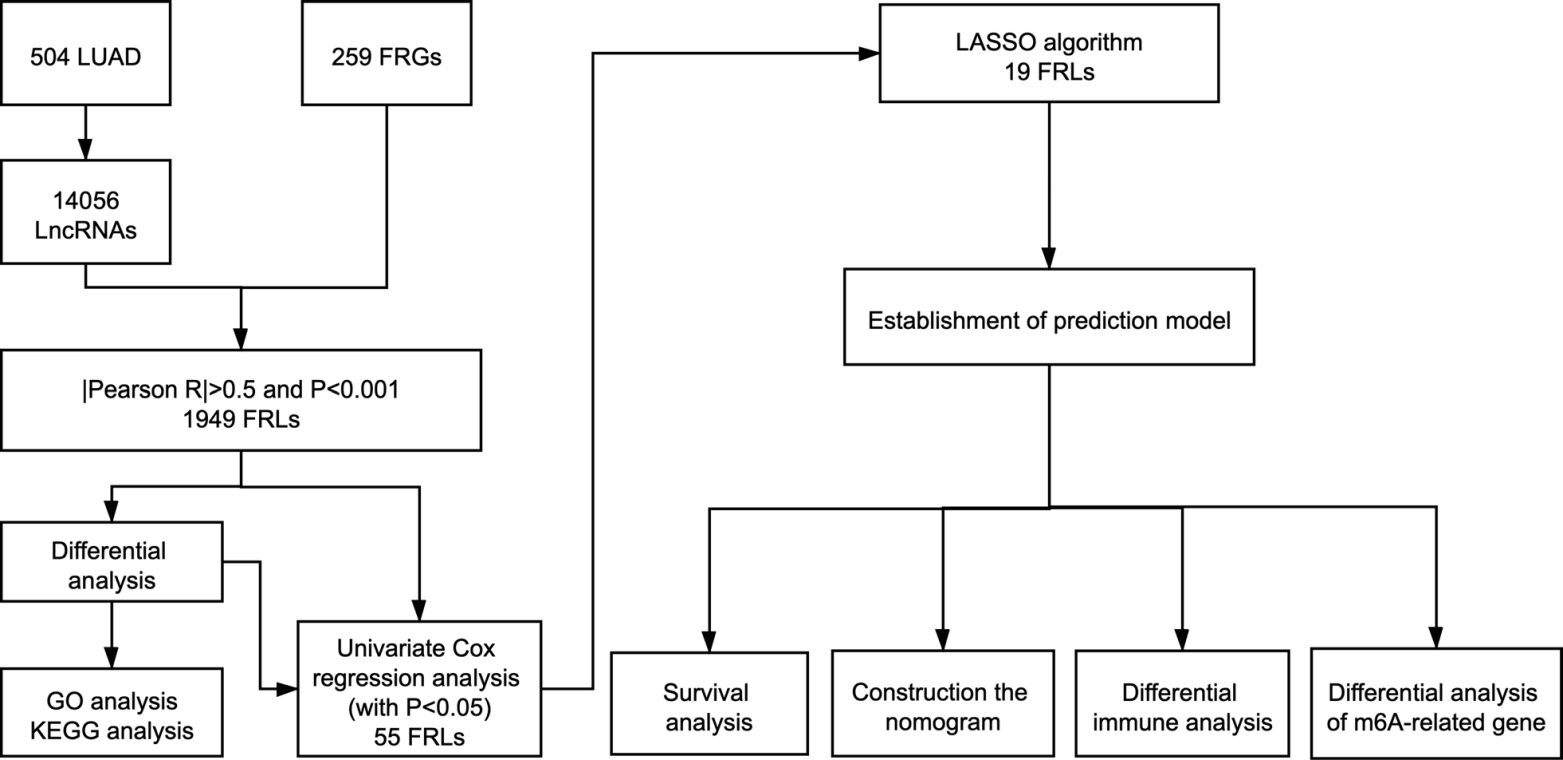
Study workflow

The study protocol was conducted in accordance with the Declaration of Helsinki. Informed consent was waived owing to the retrospective nature of this study. Transcriptome profiling data [fragments per kilobase of transcript per million mapped reads normalized] and the relevant clinical data were obtained from the Genomic Data Commons Data Portal (https://portal.gdc.cancer.gov). The inclusion criteria were as follows: (1) LUAD confirmed by pathology; (2) patients with LUAD who had complete transcriptome profiling data; and (3) patients with LUAD who had complete survival data. The exclusion criteria were as follows: incomplete information of the transcriptome profiling data and clinical data. A total of 504 patients with LUAD were included in this study from the TCGA cohort. A total of 259 FRGs (382 annotations) were included in this study, which were downloaded from the FerrDb database (http://www.zhounan.org/ferrdb) and could be divided into drivers, markers and suppressors. The annotation of the lncRNAs in the TCGA dataset was conducted by the annotation file of Genome Reference Consortium Human Build 38 (GRCh38), which was downloaded from the GENCODE website. A total of 14,056 lncRNAs were identified based on recognizing the ensemble IDs of the genes in the TCGA datasets. The flow chart of study is shown in Fig. 1.

### Ferroptosis-related lncRNA (FRL) selection and differential expression gene (DEG) analysis

FRLs were initially identified based on the expression of lncRNAs and FRGs by Pearson correlation analysis with |Pearson R|> 0.5 and *P* < 0.001. The different expression levels of FRGs and FRLs were analyzed by Wilcoxon tests between the normal group and the tumor group with log2fold change (FC) > 1 and false discovery rate (FDR) < 0.05. Gene Ontology (GO) and Kyoto Encyclopedia of Genes and Genomes (KEGG) analyses of these DEGs were performed. Then, the prognostic FRLs were determined by univariate Cox regression analysis from the significantly different expression of FRLs (P < 0.05).

### Construction of prediction model and validation

The training and test sets of patients with LUAD were randomly classified at a ratio of 1:1. The final prognostic model was constructed using the optimized subset of prognostic FRLs chosen by the least absolute shrinkage and selection operator (LASSO) algorithm. The risk score (RS) was computed by the chosen lncRNA expression weighted by their corresponding coefficients. The prognostic model was then assessed by ROC analysis and a risk plot. Using the median RS, those with LUAD were separated into either high- or low-risk groups. The OS of patients with LUAD in the two-risk group was shown by the Kaplan–Meier (KM) method in the training and test sets and the comparison was conducted. The RS and clinical features of patients with LUAD were analyzed by univariate and multivariate Cox regression analysis. The differential analysis of each clinical group based on the two-risk group was conducted by the chi-square test. The development of the nomogram was conducted based on RS and clinical features and evaluated by ROC analysis.

### Gene set enrichment analysis (GSEA) and differential immune analysis

GSEA was conducted by GSEA software (version 4.1.0) in the two risk groups of LUAD. A p value < 0.05 and the method of signal2noise were selected to denote enrichment significance. Then, the TIMER [23], CIBERSORT [24], CIBERSORT-ABS, QUANTISEQ [25], Microenvironment Cell Populations-counter (MCPCOUNTER) [26], XCELL [27] and Estimating the proportion of Immune and Cancer cells (EPIC) [28] tools were used to estimate the abundances of immune cells between the two risk groups. In addition, single sample GSEA was used to quantify the immune cells and the pathways between the two risk groups. The expression levels of immune checkpoint-related genes in the two groups were compared by the Wilcoxon test.

### Analysis of m6A-related genes

A total of 23 m6A regulators were identified based on previous literature, which could be divided into writers, erasers and readers. METTL3, METTL14, METTL16, WTAP, VIRMA, RBM15, RBM15B, and ZC3H13 were the regulators as writers. FTO and ALKBH5 were the regulators as erasers. YTHDC1, YTHDC2, YTHDF1, YTHDF2, YTHDF3, HNRNPC, FMR1, LRPPRC, HNRNPA2B1, IGF2BP1, IGF2BP2, IGF2BP3, and RBMX were the regulators as readers. The expression levels of m6A-related genes in the two-risk group were also compared.

### Statistical analysis

R statistical software (version 4.0.5) was used for all statistical analyses. Student’s t test, Mann–Whitney U test or Kruskal–Wallis H-test was used for continuous variables. A chi-square test or Fisher’s exact test was used for categorical variables. The “limma” package was used to analyze transcriptome profiling data. The “clusterProfiler”, “org.Hs.eg.db”, “enrichplot”, and “ggplot2” packages were used to conduct the GO analysis and KEGG analysis and build bar plots and bubble plots. The “survival”, “survminer”, “caret”, “glmnet” and “timeROC” packages were used to conduct the survival analysis, build the prognostic model and obtain the ROC curve. The “ggalluvial”, “ggplot2”, and “dplyr” packages were used to develop the Sankey diagram. The “pheatmap” package was used to develop heatmaps and risk plots. The “survival” and “regplot” packages were used to develop the nomogram. The “GSVA”, “GSEABase”, “ggpubr”, and “reshape2” packages were used to develop box plots for the immune function analysis and the differential expression of checkpoint-related genes and m6A-related genes between the two-risk group. A two-sided P < 0.05 was considered statistically significant.

## Results

### Identification of FRLs in LUAD patients

**Fig. 2:**
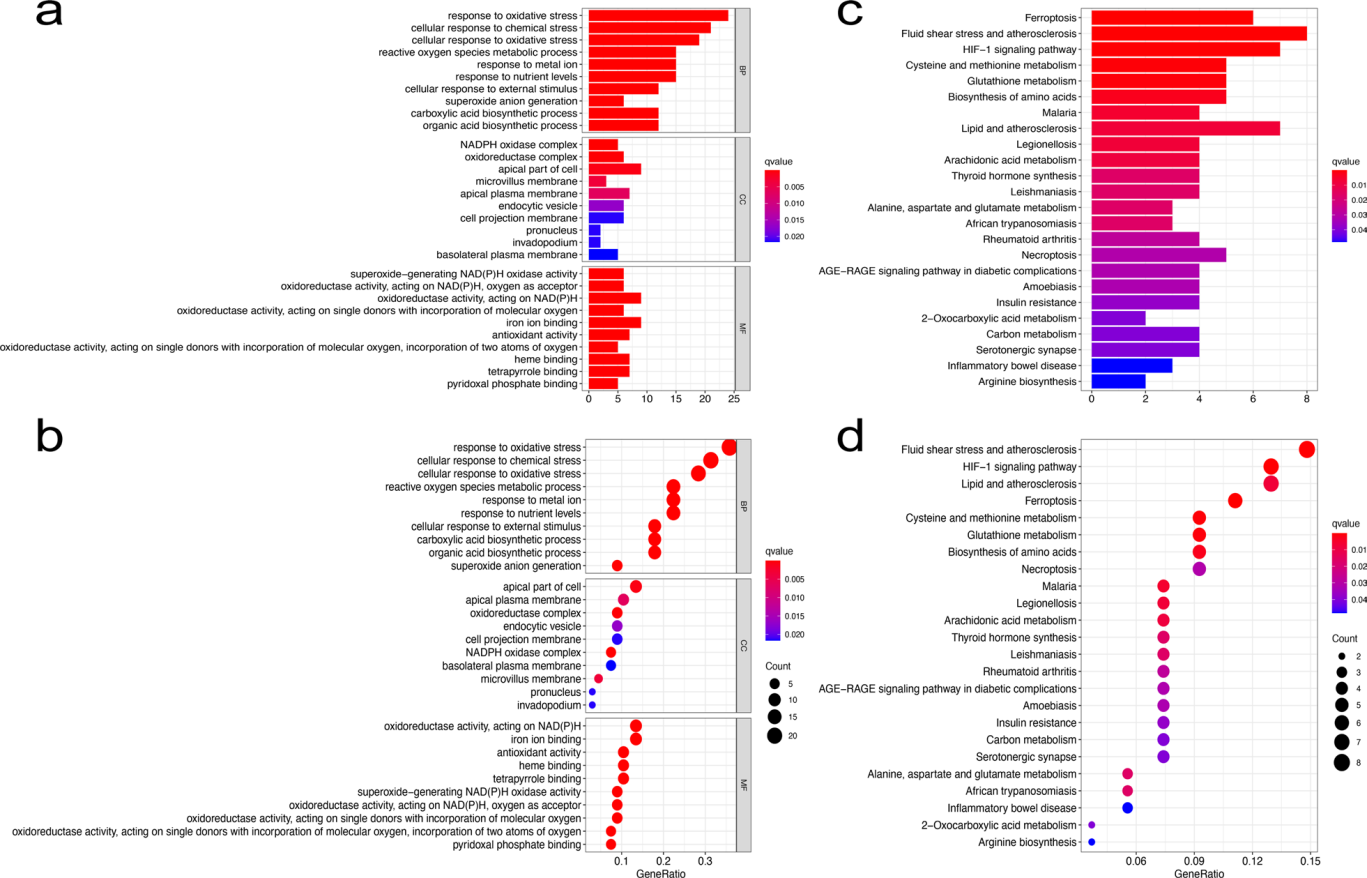
GO and KEGG analysis of differentially expressed genes (DEGs). **a**, **b** GO analysis of DEGs between the normal group and the tumor group, including biological process (BP), cellular component (CC), and molecular function (MF); **c, d**: KEGG analysis of DEGs between the normal group and the tumor group

A total of 1949 FRLs were determined by Pearson correlation analysis to be significant (with |Pearson R|> 0.5 and *P* < 0.001). The expression of 70 FRGs and 664 FRLs was significantly different between the normal group and the tumor group. With regard to biological processes, GO was used to enrich the DEGs by biological process (BP), cellular component (CC), and molecular function (MF), as shown in Fig. 2a-b. With regard to BP, these DEGs were mainly enriched in response to oxidative stress and cellular response to chemical stress. With regard to CC, these DEGs were mainly enriched in the NADPH oxidase complex. With regard to MF, these DEGs were mainly enriched in superoxide-generating NADPH oxidase activity. Furthermore, we used KEGG to conduct pathway analysis on these DEGs and found that the DEGs were mainly enriched in ferroptosis and fluid shear stress and atherosclerosis, as shown in Fig. 2c-d. Then, a total of 55 prognostic FRLs were selected by univariate Cox regression analysis (with P < 0.05).

### Establishment of predictive model and survival analysis

**Fig. 3:**
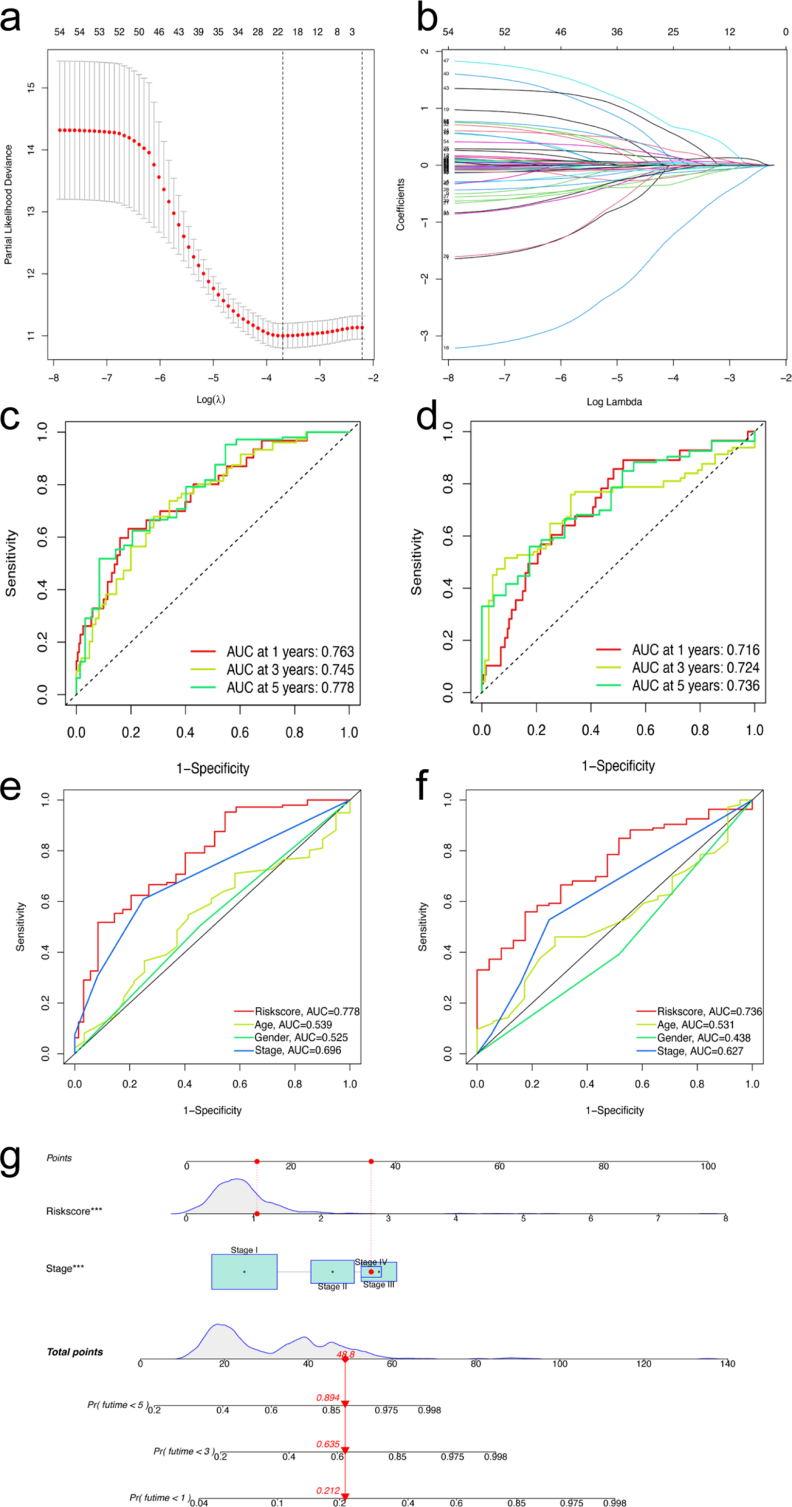
Building the prognostic model and nomogram. **a**, **b** The process of LASSO regression which added a penalty term for shrinkage of the parameter estimates to the least-squares loss function; **c**–**f** the ROC of prognostic model and clinical features for 5-years OS in the training set (**c**, **e**) and validation set (**d**, **f**); **g** the nomogram based on clinical features and prognostic model for OS of patients with LUAD. The red highlights showed that the total point of the patient with LUAD who was Stage IV was 48.8 and the probability of survival time of the patient less than 1, 3, 5 years was 0.212, 0.635, 0.894, respectively

**Fig. 4:**
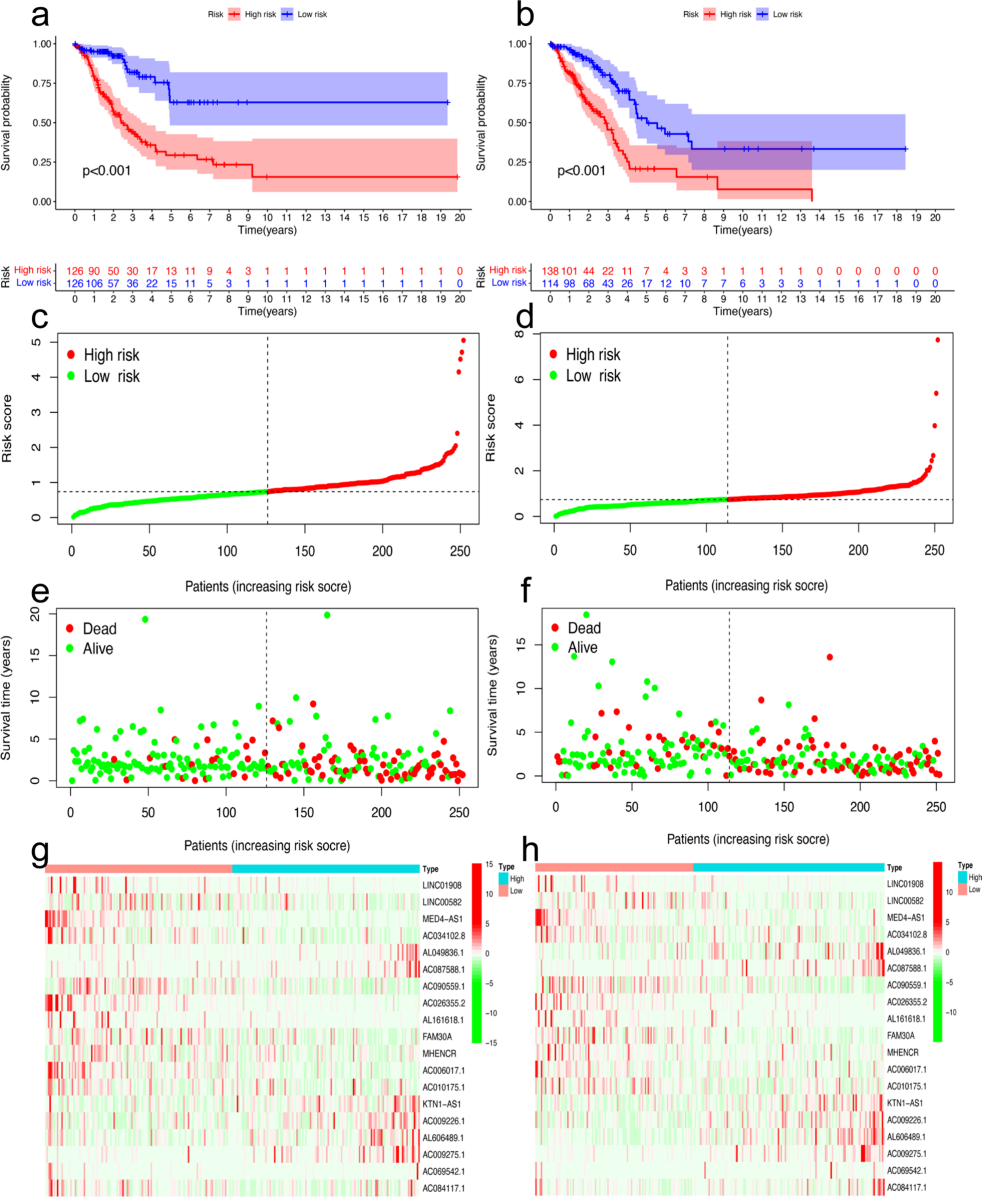
Survival analysis of LUAD based on risk group and risk plot. **a**, **b**: The figures showed that overall survival of high-risk group was significantly worse than low-risk group in the training set (**a**) and validation set (**b**); **c**, **d** Patients were divided into two risk groups by median value of the risk score in the training set (**c**) and validation set (**d**); **e**, **f** The distribution of patients with survival status based on two risk groups in the training set (**e**) and validation set (**f**); **g**, **h** The heatmap of the expression of 19 ferroptosis-related lncRNAs between two-risk group in the training set (**g**) and validation set (**h**)

**Fig. 5:**
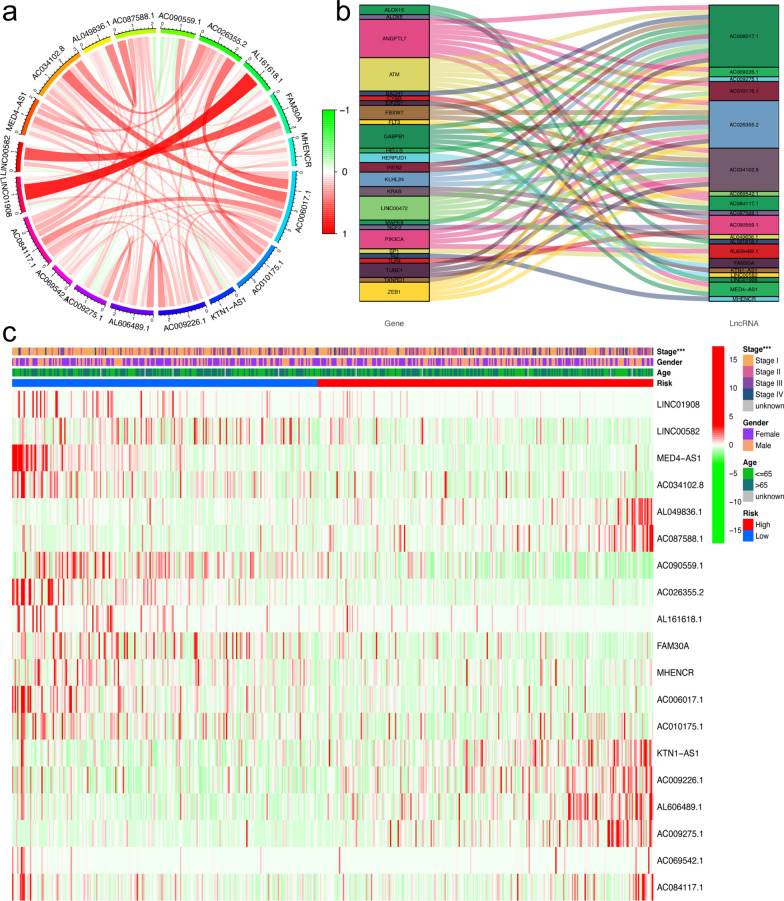
Correlation analysis based on risk group in all patients. **a** Circle map showing the optimized subset of prognostic ferroptosis-related lncRNAs (FRLs) in the coexpression network. The bar represented the correlation. The thickness of the line indicates the strength of the correlation coefficient. **b** Sankey diagram showing the coexpression of ferroptosis-related genes and 19 FRLs. **c**: The figure shows that the patients in the two-risk group were significantly different in these subgroups of patients with stage of disease. The heatmap also showed the 19 FRL expressions in the two-risk group. *P < 0.05, **P < 0.01, ***P < 0.001

The 504 patients were randomly divided into a training set and a validation set. There were no significant differences in clinical characteristics between the training and validation cohorts (Table 1). Then, 19 prognostic FRLs selected by the LASSO algorithm were used to calculate the RS, and the formula is shown below (Fig. 3a-b). RS = exp[(− 0.155689001) *LINC01908 + (− 0.048007678) *LINC00582 + (− 0.966427117) *MED4-AS1 + (− 0.060758065) *AC034102.8 + (0.043720579) *AL049836.1 + (0.104812457) *AC087588.1 + (− 0.155348446) *AC090559.1 + (− 0.110255866) *AC026355.2 + (− 0.035440534) *AL161618.1 + (− 0.064732213) *FAM30A + (− 0.01945136) *MHENCR + (− 0.240780809) *AC006017.1 + (0.390969837) *AC010175.1 + (0.210496796) *KTN1-AS1 + (0.613005188) *AC009226.1 + (0.073272911) *AL606489.1 + (0.031857583) *AC009275.1 + (0.040530768) *AC069542.1 + (0.079655558) *AC084117.1]. After the LASSO algorithm, the area under the ROC curve (AUC) of the model for 1-year, 3-year, and 5-year OS was 0.763, 0.745, and 0.778 in the training set and 0.716, 0.724, and 0.736 in the validation set, respectively (Fig. 3c-d). The ROCs of clinical features are also shown in Fig. 3e-f. After Cox regression analysis, RS and stage were both considered significant predictive factors for the OS of patients with LUAD in all groups (Table 2). Then, a nomogram based on RS and stage was established, and an ROC curve was drawn (Fig. 3g, Additional file 1). According to the median value of the RS, the survival status was shown based on the two risk groups, and the OS of the high-risk group was significantly poorer than that of the low-risk group in both the training set (P < 0.001) and test set (P < 0.001), as shown in Fig. 4a-h. The optimized subset of prognostic FRLs in the coexpression network and the relationship with FRGs were visualized using the circle map and Sankey diagram (Fig. 5a-b). The differential analysis of each clinical feature in the two risk groups is shown in the heatmap, and the stage of patients with LUAD was significantly different (Fig. 5c).

**Table 1:** Clinical information of 504 patients with LUAD from the TCGA database

Clinical features	TCGA database (n = 504)	Training group (n = 252)	Validation group (n = 252)
Age	65.30 ± 10.03	65.26 ± 9.91	65.34 ± 10.17
Sex			
Male	234	122	112
Female	270	130	140
Pathological stage			
I	270	137	133
II	119	62	57
III	81	38	43
IV	26	12	14
Unknown	8	3	5
T			
T1	168	77	91
T2	269	137	132
T3	45	25	20
T4	19	11	8
Unknown	3	2	1
N			
N0	325	162	163
N1	94	50	44
N2	71	30	41
N3	2	1	1
Unknown	12	9	3
M			
M0	335	163	172
M1	25	12	13
Unknown	144	77	67

**Table 2:** Univariate and multivariate Cox regression analysis

Variable	Univariate Cox regression		Multivariate Cox regression	
	HR (95% CI)	*P* value	HR (95% CI)	*P* value
Training cohort				
Age	1.013 (0.990–1.036)	0.261	1.032 (1.007–1.057)	0.011
Sex	1.444 (0.935–2.228)	0.097	1.317 (0.849–2.043)	0.218
Stage	1.763 (1.431–2.171)	< 0.001	1.803 (1.435–2.266)	< 0.001
Risk score	2.405 (1.952–2.964)	< 0.001	2.444 (1.964–3.040)	< 0.001
Test cohort				
Age	1.004 (0.983–1.024)	0.734	1.005 (0.985–1.025)	0.631
Sex	0.867 (0.575–1.308)	0.497	0.837 (0.544–1.287)	0.417
Stage	1.526 (1.265–1.842)	< 0.001	1.557 (1.285–1.887)	< 0.001
Risk score	1.718 (1.435–2.056)	< 0.001	1.734 (1.438–2.093)	< 0.001

CI: confidence interval; HR, Hazard ratio

### GSEA

**Fig. 6:**
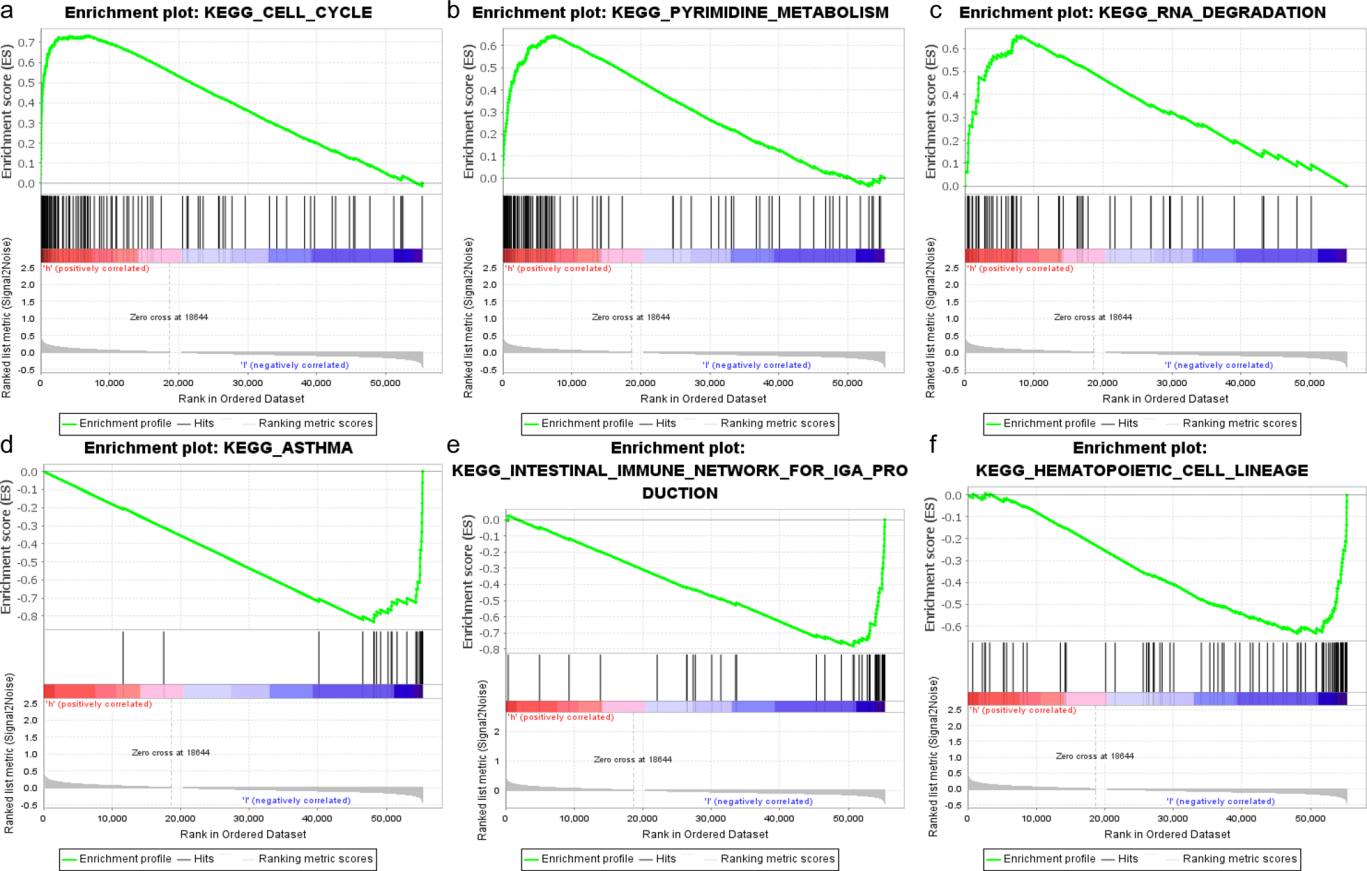
Gene set enrichment analysis (GSEA) with two-risk group. **a**–**c** The top 3 gene sets enriched in the high-risk group included the cell cycle (NES = 2.31, Norm P < 0.001, FDR q = 0.003), pyrimidine metabolism (NES = 2.26, Norm P < 0.001, FDR q = 0.003), RNA degradation (NES = 2.23, Norm P < 0.001, FDR q = 0.002); **d**–**f** The top 3 gene sets enriched in the low-risk group included asthma (NES = − 2.05, Norm P < 0.001, FDR q = 0.041), intestinal immune network for IgA production (NES = − 2.01, Norm P = 0.004, FDR q = 0.034) and hematopoietic cell lineage (NES = − 1.95, Norm *P* < 0.001, FDR q = 0.047)

GSEA revealed that the set of genes was enriched in the cell cycle, pyrimidine metabolism and RNA degradation for the high-risk group (Fig. 6a-c). In the low-risk group, the gene set was mainly enriched for asthma, intestinal immune network for IgA production and hematopoietic cell lineage (Fig. 6d-f).

### Differential immune analysis of RS

**Fig. 7:**
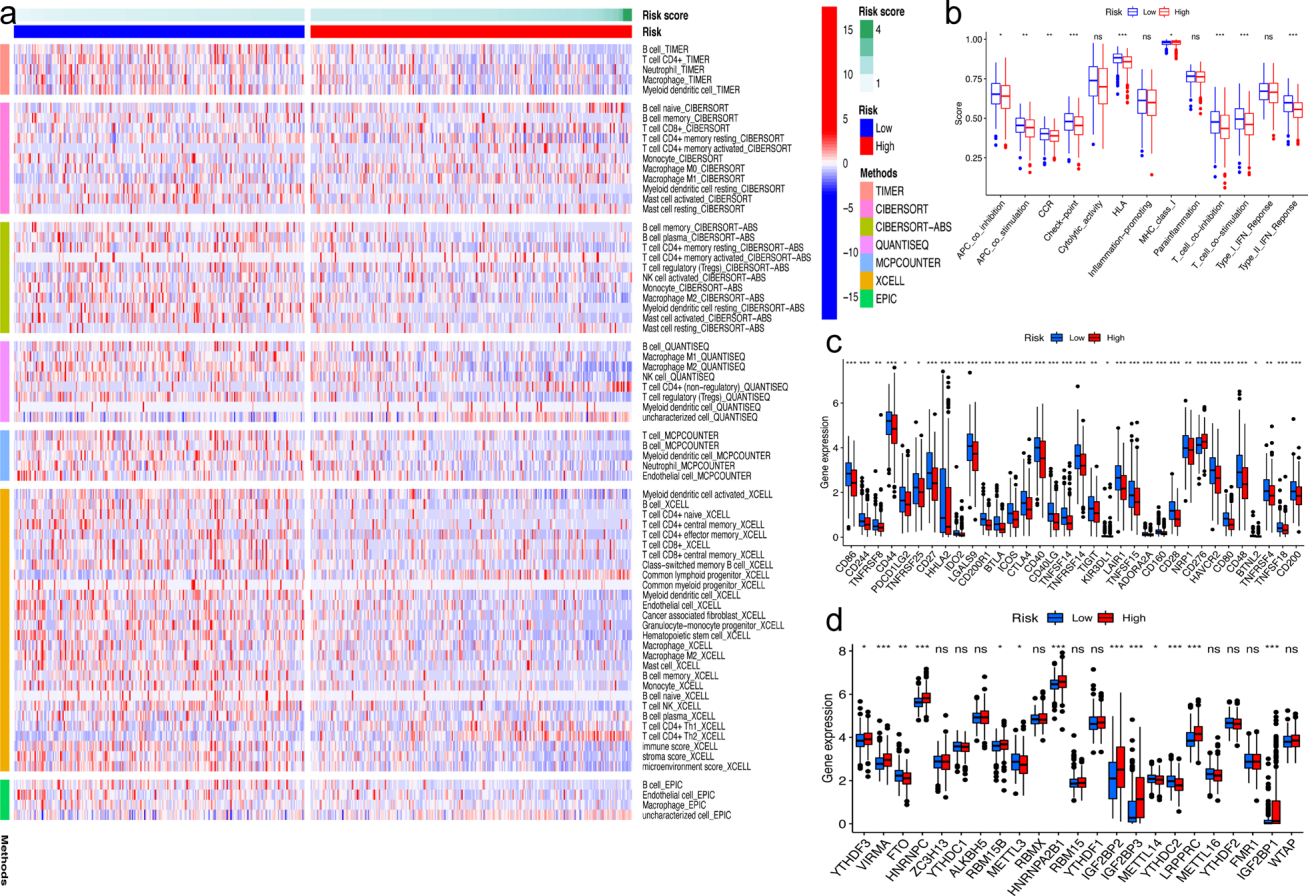
Immune-heatmap analysis and differential analysis. **a** Immune heatmap showing the abundances of immune cells between two risk groups by 7 kinds of tools; **b** boxplot showing the difference score of 13 kinds of immune function between the two-risk group; **c** boxplot showing the difference in the expression of 34 checkpoint genes between the two-risk group; **d** boxplot showing the difference in the expression of 23 m6A-related genes between the two-risk group. *P < 0.05, **P < 0.01, ***P < 0.001

A heatmap of immune infiltration based on the TIMER, CIBERSORT, CIBERSORT-ABS, QUANTISEQ, MCPCOUNTER, XCELL and EPIC tools is shown in Fig. 7a. The results of the comparative analysis of immune function between the two risk groups are shown by the boxplot (Fig. 7b). The expression of 34 checkpoint genes was significantly different between the two risk groups (Fig. 7c).

### Differential expression analysis of m6A

The difference in the expression level of m6A-related genes between the two risk groups is shown in Fig. 7d. The expressions of 13 m6A-related genes were significantly different between two risk groups. The expression levels of VIRMA, HNRNPC, HNRNPA2B1, IGF2BP1, IGF2BP2, IGF2BP3 and LRPPRC were significantly higher in the high-risk group (P < 0.001). The expression level of YTHDC2 was significantly lower in the high-risk group (P < 0.001).

## Discussion

After univariate Cox regression analysis and the LASSO algorithm, 19 prognostic FRLs were established in the model for predicting the OS of patients with LUAD. Those with LUAD were separated by the median value of the RS. The high-risk group showed significantly worse OS than the low-risk group (P < 0.001). Univariate and multivariate Cox regression revealed that a valuable significant risk factor was RS. The results of the differential immune analysis indicated that the RS was correlated with immune cell infiltration in our study. In addition, the differential expression of m6A-related genes in the two-risk group could indicate that FRLs were associated with these m6A-related genes in the prognosis of patients with LUAD. This study is the first to comprehensively analyze FRLs in the prognosis of patients with LUAD, immune cell infiltration and m6A modification.

Many studies have shown that lncRNAs can regulate ferroptosis in the oncogenesis and progression of malignant tumors [29–31]. A study by Zhang et al. showed that OIP5-AS1 could promote cell growth and inhibit ferroptosis in prostate cancer, which functioned as a competing source of endogenous RNAs (ceRNAs) for miR-128-3p to regulate the expression of SLC7A11[29]. A study by Mao et al. showed that P53RRA could lead to ferroptosis by retaining p53 in the nucleus and serves as a tumor suppressor by displacing p53 from a cytosolic G3BP1 complex [30]. A study by Wu et al. showed that NEAT1 could modulate the expression of ACSL4 in NSCLC, which was a FRL in previous reports, and further affect the ferroptosis process [31]. These studies indicated that FRLs could contribute to the tumorigenesis and progression of malignant tumors, regulate ferroptosis and influence the invasion of tumors. However, few studies have examined how FRLs affect the progression of LUAD. In this study, the gene set of FRLs from patients with LUAD was mainly enriched in the cell cycle, pyrimidine metabolism and RNA degradation in the high-risk group, which had significantly worse OS than the low-risk group. These results indicated that FRLs could affect the progression of patients with LUAD and the OS of patients with LUAD.

In our study, 19 prognostic FRLs were confirmed to build the prediction model of patients with LUAD. A total of 8 out of 19 FRLs, which included LINC00582, MED4-AS1, AC090559.1, AL161618.1, FAM30A, MHENCR, KTN1-AS1 and AL606489.1, were previously investigated in tumors [32–39]. The results of both Wu et al. and Guo et al. showed that the expression of AC090559.1 was higher and the expression of AL606489.1 was lower in the low-risk group, and these two lncRNAs were both considered significant predictive factors for the OS of patients with LUAD [32, 33]. These findings are in line with the results of our study. The study of Li et al. showed that KTN1-AS1 expression was upregulated in NSCLC tissue and was positively correlated with poor prognosis, which could be considered a ceRNA for miR-130a-5p to regulate NSCLC cell growth and apoptosis [34]. These findings are also in line with the results of our study. In addition, Lima et al. showed that the expression of FAM30A was positively associated with the level of antibody titers and the lncRNA might regulate immunoglobulin heavy locus gene segments [35]. Furthermore, several studies have shown that abnormal immune cells contribute to the progression of LUAD and regulate ferroptosis, and checkpoint inhibitor-based immunotherapies have improved the survival of patients with advanced malignancies [40–44]. In our study, significant differences in immune cell infiltration and function and the expression of immune checkpoints between the two risk groups were also confirmed to a certain extent. A parallel study by Yao et al. identified 7 FRLs that had diagnostic utility in OS for patients with LUAD, and the results showed that the AUCs of the risk model based on 7 FRLs for 1 year, 2 years, and 3 years were 0.711, 0.658, and 0.676 in the training set and 0.593, 0.577, and 0.525 in the validation set [45]. A net of 7 FRL differences was determined in their study, while a net of 19 FRL differences was determined in our study. What’s more, stage III had a slightly higher point than stage IV in the nomogram of our study. The reason may be the differences between several sample sets and the TCGA cohort and insufficient sample size. However, overwhelming evidence suggests that the identified FRLs hold high diagnostic utility in the oncogenesis of LUAD, and further studies along this direction are needed.

On a separate note, several studies have investigated the relationship between m6A modification and the prognosis of LUAD [22, 46, 47]. A study by Ma et al. demonstrated that YTHDC2, a m6A-related gene, could suppress HOXA13 by m6A modification to suppress SLC3A2 and further promote ferroptosis in LUAD [22]. In addition, a study by Xu et al. demonstrated that the risk model, which was considered an independent indicator for the prognosis of LUAD, was composed of 12 m6A-related lncRNAs, and the AUC of the risk model was 0.759 [46]. It is evident that modification of m6A was able to influence the progression of cancers. Our study showed that the expression levels of 13 m6A-related genes between the two-risk group were significantly different. The results indicated that m6A modification could play a potential role in FRLs in the prediction of the prognosis of patients with LUAD, and further research is needed to obtain more details about undying associations.

While significant findings were reported above, limitations to these findings are described below. First, the use of independent LUAD cohorts or multiple databases would increase the diagnostic power and improve the nomogram over a single TCGA dataset. Second, in vitro and in vivo experiments should be used in conjunction to determine the mechanistic function of FRLs in oncogenesis.

## Conclusions

In summary, this study determined the prognostic value of FRLs in LUAD patients and used these findings to generate a prognostic model to effectively predict the OS of patients with LUAD. Moreover, a nomogram for a predictive prognostic model was built, and the differential expression of m6A-related genes in the two-risk group was confirmed. As such, the FRLs identified herein may suggest therapeutic targets for the treatment of LUAD.

## Supplementary Information

**Additional file 1.** The ROC of nomogram.

**Additional file 2.** The data analysis.

## Data Availability

All data, including the transcriptome profiling normalization data and the relevant clinical features, were acquired from the Genomic Data Commons Data Portal (https://portal.gdc.cancer.gov) of TCGA, which is the open access data. The data analysis was also included in the Additional file 2.

## Abbreviations

AUC: Area under the receiver operating characteristic curve
BP: Biological process
CC: Cellular component
CI: Confidence interval
DEGs: Differential expression genes
EPIC: Estimating the proportion of immune and cancer cells
FDR: False discovery rate
FRGs: Ferroptosis-related genes
FRLs: Ferroptosis-related long noncoding RNAs
GO: Gene ontology
GRCh38: Genome Reference Consortium Human Build 38
GSEA: Gene set enrichment analysis
HR: Hazard ratio
KEGG: Kyoto Encyclopedia of Genes and Genomes
KM: Kaplan–Meier
LASSO: Least absolute shrinkage and selection operator
lncRNAs: Long noncoding RNAs
LUAD: Lung adenocarcinoma
m6A: N6-methyladenosine
MCPCOUNTER: Microenvironment cell populations-counter
MF: Molecular function
NES: Normalized enrichment score
Norm: Normalized
OS: Overall survival
ROC: Receiver operating characteristic
RS: Risk score
TCGA: The Cancer Genome Atlas
