# Supplementary figures and images for: Risk stratification of lung adenocarcinoma using a nomogram combined with ferroptosis-related LncRNAs and subgroup analysis with immune and N6-methyladenosine modification
Source: BMC Med Genomics. 2022 Jan 29;15:15. doi: 10.1186/s12920-022-01164-5 (PMC8800367; doi:10.1186/s12920-022-01164-5)

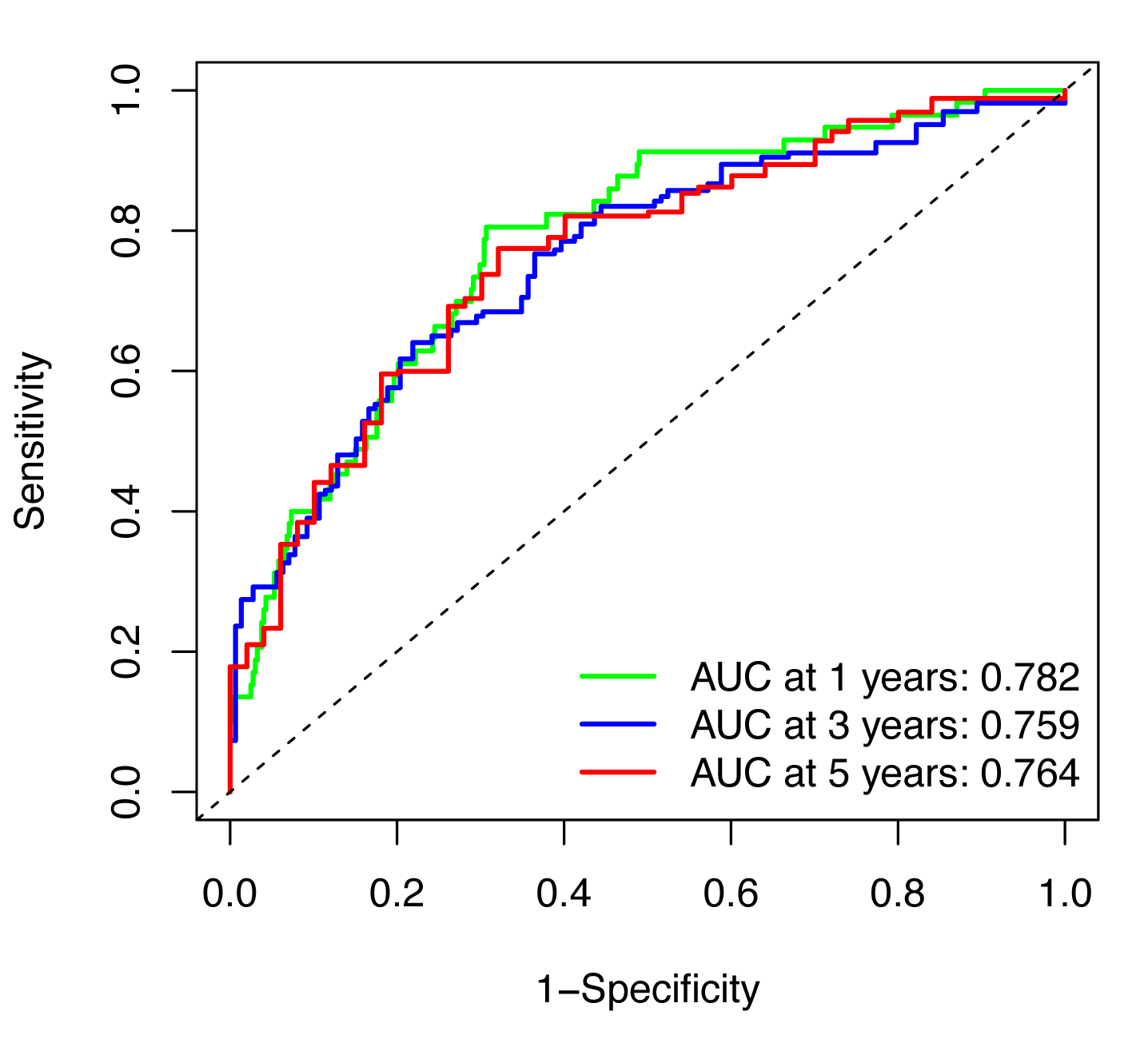

Supplement: Supplementary file 1 — Additional file 1. The ROC of nomogram. [file 12920_2022_1164_MOESM1_ESM.tif]
